# APOA-I: A Possible Novel Biomarker for Metabolic Side Effects in First Episode Schizophrenia

**DOI:** 10.1371/journal.pone.0093902

**Published:** 2014-04-07

**Authors:** Xueqin Song, Xue Li, Jinsong Gao, Jingping Zhao, Youhui Li, Xiaoduo Fan, Luxian Lv

**Affiliations:** 1 Department of Psychiatry, The First Affiliated Hospital of Zhengzhou University, Zhengzhou, China; 2 Henan Key Lab of Biological Psychiatry, Xinxiang Medical University, Xinxiang, China; 3 University of Massachusetts Medical School UMass Memorial Medical Center, Worcester, Massachusetts, United States of America; 4 The Mental Health Institute of the Second Xiangya Hospital,Central South University, Changsha, Hunan, China; 5 Department of Psychiatry, Henan Mental Hospital, The Second Affiliated Hospital of Xinxiang Medical University, Xinxiang, China; Queensland Institute of Medical Research, Australia

## Abstract

The purpose of this study was to investigate the change in plasma protein expression in first episode schizophrenia after an 8-week treatment with risperidone, and to explore potential biomarkers for metabolic side effects associated with risperidone treatment. Eighty first-episode schizophrenia patientswere enrolled in the study. Fifteen of the 80 patients were randomly selected to undergo proteomic analysis. Plasma proteins were obtained before and after the 8-week risperidone treatment, and measured using two-dimensional gel electrophoresis (2-DE), Matrix-Assisted Laser Desorption/Ionization Time of Flight Mass Spectrometry(MALDI-TOF/TOF) and peptide mass fingerprinting.Proteins with the highest fold changes after risperidone treatment were then measured for all 80 patients using enzyme linked immunosorbent assay (ELISA). The relationship between changes in plasma protein levels and changes in metabolic parameters after risperidone treatment was examined. In 15 randomly selected patients, approximately 1,500 protein spots were detected in each gel by 2-DE. Of those proteins, 22 spots showed significant difference in abundance after risperidone treatment (p's<0.05). After MALDI-TOF peptide mass fingerprinting, apolipoprotein A-I (APOA-I) and Guanine Nucleotide Binding Protein, Alpha Stimulating (GNAS), were found to have the highest fold changes.The content of APOA-I was significantly increased, and the content of GNAS was significantly decreased after risperidone treatment (p's<0.05). The analysis in the entire study sample showed similar findings in changes of APOA-I and GNAS after risperidone treatment. Further analysis showed significant relationships between changesin APOA-1 and changes in triglyceride, total cholesterol, and body mass index after controlling for age, gender and family history of diabetes. Similar analysis showed a trend positive relationship between changes in GNAS and changes in BMI. Using proteomic analysis, the study suggested that APOA-I might be a novel biomarkers related to metabolic side effects in first episode schizophrenia treated with risperidone.

## Introduction

Risperidone is a commonly used agent for schizophrenia treatment. However, risperidone is associated with significant metabolic side effects, including weight gain [1], impaired glucose tolerance and elevated lipid levels [2]–[4]. These adverse reactions increase the risk for cardiovascular disease and reduce treatment compliance. The mechanism of metabolic problems related to antipsychotic treatment remains largely unknown at this time.

Proteomics uses a large-scale, automatic analysis of cellular protein expression profiles with both high-throughput and high-sensitivity. Proteomic analysis has been widely used to identify biomarkers for the diagnosis and treatment response of various diseases [5]. Previous studies using proteomic analysis have identified several proteins that are possible novel biomarkers related to the development of metabolic problems, such as obesity, diabetes and elevated lipids, in the general population [6]–[8].

Using proteomic analysis, the present study was to investigate the change in plasma protein expression in first episode schizophrenia after an 8-week treatment with risperidone, and to identify potential biomarkers for metabolic side effects associated with risperidone treatment.

## Materials and Methods

### Subjects

The study was carried out in accordance with The Code of Ethics of the World Medical Association (Declaration of Helsinki) for experiments involving humans. This study was conducted at the inpatient units of Henan Mental Hospital between January 2011 and December 2011. All subjects gave their written informed consent prior to participation in this study after fully understood the purpose and procedures. The patients' capacity to sign the informed consent was based on the judgment of their clinician. If a patient was unable to give informed consent, it was signed by his/her caregiver. All study protocols were approved by the Institutional Review Board of Henan Mental Hospital. Inpatients with first episode schizophrenia were recruited from Henan Mental Hospital. The diagnosis of schizophrenia was determined by a research psychiatrist (X.L.) using the Structured Clinical Interview for DSM-IV Axis I Disorders. The inclusion criteria included: 1) age 18 to 45 years; 2) disease duration less than 2 years. Patients were excluded if they had: 1) prior antipsychotic treatment; 2) previous history of alcohol or other substance use; 3) additional psychiatric diagnosis other than schizophrenia; 4) certain medical conditions including acute infection, heart disease, epilepsy, hepatic or renal diseases, diabetes, aplastic anemia, asthma or autoimmune diseases; 5) female patients who were pregnant or lactating. A complete medical history was obtained from all subjects. All subjects were treated in the same hospital, underwent daily physical examination and weekly routine laboratory tests. All patients had the same standard diet provided by the hospital, and structured daily light-to-moderate physical activity (30 minutes, led by nursing staff).

After baseline assessment, all patients were treated with risperidone with the dose ranging from 4 mg to 6 mg per day based on the clinical judgment of treating psychiatrists. No other medication was allowed during the study except benzodiazepines for insomnia and anticholinergic agents for dystonia reaction.

### Assessment

Symptoms of schizophrenia were assessed using the Positive and Negative Syndrome Scale (PANSS). The PANSS was assessed before and after 8 weeks of risperidone treatment. Family history of psychiatric disorders including both Axis I and Axis II disorders was obtained from both patients and their family members if they were available. For all subjects, weight (kg), height (m), plasma glucose (mmol/l) and lipid levels (mmol/l) were measured before and after treatment. Body mass index (BMI) (kg/m^2^) was calculated based on weight and height. Glucose oxidase was used to measure plasma glucose level. Triglycerides, cholesterol, LDL and HDL levels were measured by the enzymatic colorimetric method. Fifteen out of the 80 subjects were randomly selected for proteomic analysis (8 females and 7 males).

### Sample preparation

Venous blood (10 mL) was collected in two 5 ml BD vacutainer between 07:00 and 08:00 to avoid circadian fluctuation of measured parameters. One of the BD vacutainer was an EDTA anticoagulant tube. The blood in EDTA anticoagulant tube was centrifuged immediately after being collected. After centrifugation (3000 rpm, 10 min), plasma was separated from the supernatant, and then stored at −70°C for proteomic analysis. The rest 5 ml blood was used to measure plasma levels of glucose and lipid.

### Depletion of high-abundance plasma proteins

Plasma samples (500 μL) were processed to remove those most abundant plasma proteins (i.e., albumin, IgG, IgM, IgA, transferrin, fibrinogen, alpha2-macroglobulin, alpha1-anti-trypsin, haptoglobin) using antibody-based immunodepletion spin columns in accordance with the manufacturer's protocol.

### Two-dimensional gel electrophoresis

Two-dimensional gel electrophoresis was performed as described previously [9]. Three replicates were done for each sample. Image Master 2-D Platinum software (GE Amersham) was used for the analysis of silver–stained gels. Each paired spot was manually verified to ensure a high level of reproducibility between normalized spot volumes of gel produced in triplicate data. The overlapping measure ratio was chosen to examine change in protein expression; a change of 1.2-fold or greater [10], [11] was considered significant.

### Image analysis – determination of quantitative differences

Protein spots were cut and destained from preparative gels. Mass spectrography (MS) and MS/MS spectra were obtained using the ABI 4800 Proteomics Analyzer Matrix-Assisted Laser Desorption/Ionization Time of Flight Mass Spectrometry (MALDI-TOF/TOF) (Applied Biosystems, Foster City, CA), which operates in a result-dependent acquisition mode. The GPS Explorer software (Applied Biosystems, Foster City, CA) and MASCOT (Matrix Science, London, UK) were utilized with the following parameter: trypsin cleavage, one missed cleavage allowed; carbamido methylation, fixed modification; oxidation of methionines, variable modification; peptide mass tolerance, 100 ppm; fragment tolerance, ±0.3 Da. The minimum ion score confidence interval for MS/MS data was set as 95%.

### Protein identification by peptide mass fingerprinting

Acquired MS/MS spectra were searched against the NCBI database (Dec 16th, 2006) using an in-house version of MASCOT search engine 2.1 (Matrix Sciences, London, UK). The mass accuracies of the precursor and fragment ions were 150 ppm and 0.7 Da, respectively. The proteins with the highest fold changes were considered for further analysis. Using this criterion, apolipoprotein A-1(APOPA-I) and Guanine Nucleotide Binding Protein, Alpha Stimulating (GNAS) were selected, and were further analyzed using enzyme linked immunosorbent assay (ELISA) for the entire study sample to validate the results from proteomic analysis for 15 randomly selected subjects.

### ELISA for APOA-I and GNAS for the entire study sample

ELISA was used to measure levels of APOA-I and GNAS for all 80 subjects. The identity of subject was blinded using a code number. Levels of APOA-I and GNAS in 100 μl medium were measured (APOA-I: CELL BIOLABS, STA-362,detection limit: 50 pg/ml, Intra-Assay Precision 5.2%, Inter-Assay Precision 8.4%; GNAS: Antibodies-Online, ABIN840436, detection limit: 0.078 ng/ml, Intra-Assay Precision 8%, Inter-Assay Precision 10%). Duplicate samples before and after treatment were used for quantification.

### Statistical analysis

The data were analyzed using SPSS (version 17.0; SPSS Inc., Chicago, IL). Demographics, clinical measures and laboratory values were reported using descriptive statistics. Group comparisons were performed using the Student's *t*-test. Pearson correlation coefficients were used to examine the relationships between APOA-1/GNAS and other variables of interest. For all statistical analyses, a *p* value of less than 0.05 (2-tailed) was used for statistical significance.

## Results

80 patients were enrolled in the study. None of them had missing value. Demographics of the study subjects are shown in Table 1. Clinical and laboratory measures before and after 8-week risperidone treatments are shown in Table 2. There were significant differences in the PANSS total and subscale scores, body mass index, levels of triglyceride, total cholesterol and HDL before and after treatment (p's<0.01). However, there were no significant differences in levels of plasma glucose and LDL before and after treatment (p's>0.1).

**Table 1:** Demographic characteristics of the study sample (N = 80).

	Mean±SD	Range
Age(years)	24±5	19–36
Disease duration (months)	11±5	1–23
Age of illness onset (years)	23±4	18–34
	N	%
Gender		
Male	42	52.5
Female	38	47.5
Family history of psychiatric disorders		
Yes	7	8.75
No	73	91.25
Benzodiazepine		
Yes	9	11.25
No	71	88.75

**Table 2:** Clinical measures before and after 8 weeks of risperidone treatment (N = 80).

	Before treatment	After treatment	*t* value	*p* value
Weight (Kg)	62.05±10.53	64.06±10.17	−8.90	<0.001
BMI (kg/m^2^)	22.24±1.86	22.99±1.8	−9.08	<0.001
blood glucose (mmol/l)	4.74±0.45	4.76±0.43	−1.44	0.124
triglyceride (mmol/l)	0.79±0.20	0.81±0.16	−3.11	0.001
cholesterol (mmol/l)	4.45±0.48	4.48±0.43	−3.49	0.001
LDL (mmol/l)	2.07±0.50	2.11±0.47	−1.33	0.189
HDL (mmol/l)	1.27±.015	1.26±0.15	2.05	0.043
PANSS total	71.83±7.18	53.09±8.00	28.47	<0.001
PANSS positive	20.78±4.97	13.71±2.89	13.30	<0.001
PANSS negative	15.00±4.43	10.64±2.12	9.88	<0.001
PANSS general	36.05±8.15	28.90±8.21	10.14	<0.001

BMI: body mass index; PANSS: Positive and Negative SyndromeScale.

### Changes in plasma proteins after risperidone treatment: 2-DE

**Figure 1:**
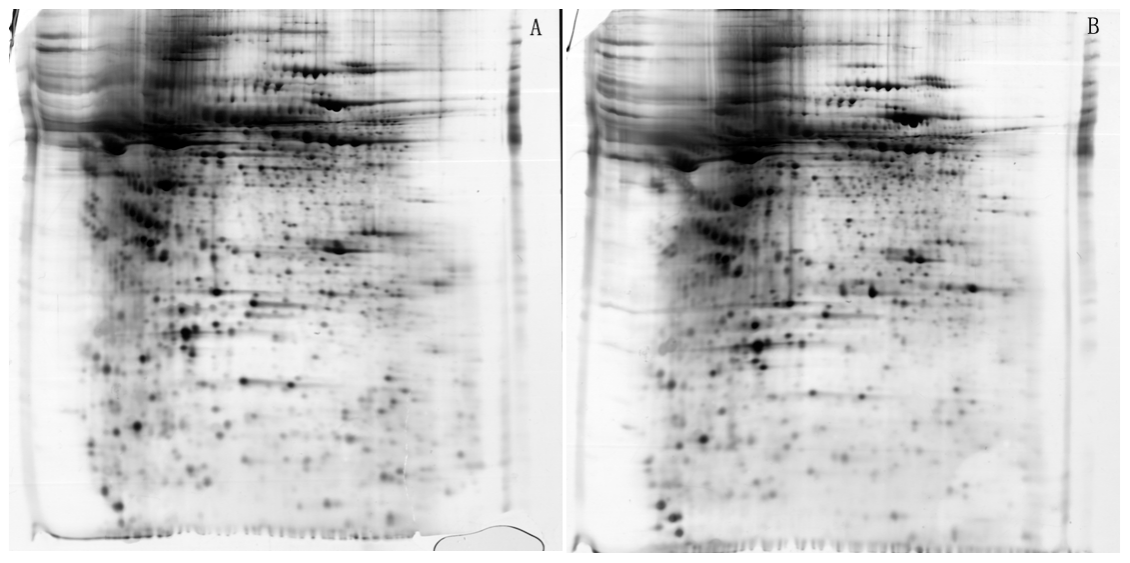
Analysis of plasma proteins before and after risperidone treatment using 2-DE. 1st-dimension: separation of isoelectric focusing (IEF), immobilized pH gradient (IPG) strips PH3-10, non-linear. 2nd-dimension: SDS-polyacrylamide gels (SDS-PAGE), 18 cm 8–16% gradient gels. Proteins were visualized using silver staining. A: Before treatment. B: After treatment.

Utilizing 2-DE, we mapped out plasma proteins individually from blood samples obtained before and after treatment in 15 randomly selected subjects. Demographic information for the patients selected for gel analysis was summarized in Table 3. More than 1500 protein spots were detected and localized in the range of pI 3–10 with a relative molecular mass of 10–70 kDa in each gel (Figure. 1A). With initial normalization of the spot intensities and subsequent statistical *t*-test analysis, 22 protein spots were found with significant differences in abundance before and after treatment (p's<0.05) (Figure 1B). These 22 proteins were then selected for MALDO-TOF/TOF analysis.

**Table 3:** Demographic characteristics of the patients selected for gel analysis (N = 15).

	Mean±SD	Rage
Age(years)	24±5	19–36
Disease duration (months)	10±5	2–21
Age of illness onset (years)	23±5	19–34
	N	%
Gender		
Male	7	46.7
Female	8	53.3
Family history of psychiatric disorders		
Yes	1	6.7
No	14	93.3
Benzodiazepine		
Yes	1	6.7
No	14	93.3

### Changes in plasma proteins after risperidone treatment: MALDI-TOF

**Figure 2:**
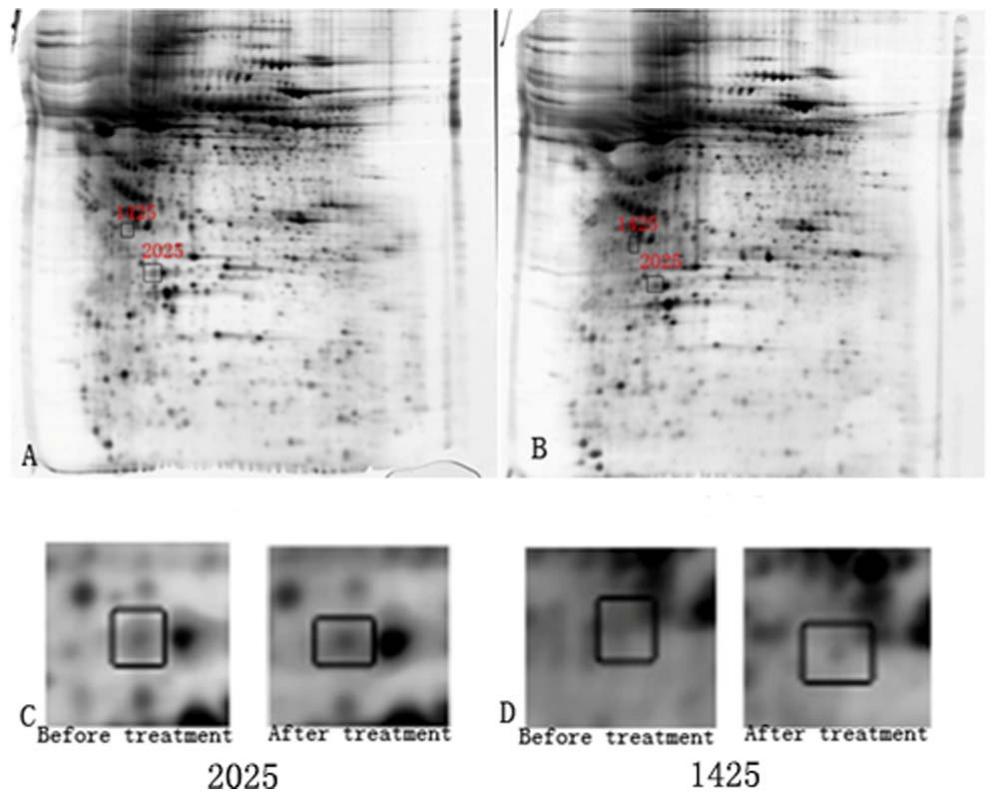
Plasma APOA-1 and GNAS expression before and after risperidone treatment using 2-DE. A: APOA-1 (spot ID 2025) and GNAS (spot ID 1425) expression before treatment. B: APOA-1 (spot ID 2025) and GNAS (spot ID 1425) expression after treatment. C: Plasma level of APOA-1 (spot ID 2025) was significantly increased after 8 weeks of risperidone treatment. D: Plasma level of GNAS (spot ID 1425) was significantly reduced after 8 weeks of risperidone treatment.

Among 22 proteins selected by 2-DE, 18 proteins successful passed the MALDI-TOF. There were 12 proteins up-regulated and 6 proteins down-regulated after treatment. The name of each protein was identified using peptide mass fingerprinting (Table 3). From these 18 proteins, two proteins with the largest fold change were identified: APOA-I (spot ID 2025, fold change 2.36278, up-regulated) and GNAS (spot ID 1425, fold change −1.84374, down-regulated) (Table 4, Figure 2).

**Table 4:** Proteins up- or down- regulation after 8 weeks of risperidone treatment.

Spot ID	Protein name	Gene name	MW(th)	PI(th)	Protein Score	Fold change	*P value*
2025	ApolipoproteinA1	APOA1	28005.3	5.8	444	2.36278	0.027
1942	Complement component 4B preproprotein	C4B	194170.1	6.89	63	1.81791	0.006
1496	Complement factor B	CFB	66697.6	5.97	428	1.56891	0.023
2011	Nebulin	NEB	14573.8	8.83	74	1.56891	0.014
1888	Complement component C8 beta chain	C8B	68714.1	8.5	202	1.44687	0.033
2089	Isoform1 of zinc finger protein185	ZNF185	74392.5	6.68	50	1.41669	0.015
1302	Plasminogen	PLG	93247.2	7.04	79	1.41550	0.030
1432	Hemopexin	HPX	28616.9	6.38	391	1.40934	0.028
1389	Histamine N-methyltransferase isoform 3	HNMT	14426.3	9.59	30	1.39999	0.031
2201	Interferon-induced guanylate-binding protein 1	GBP1	68400.9	5.97	43	1.39564	0.042
2434	Isoform gamma-A of Fibrinogen gamma chain	FGA	50092.2	5.7	57	1.33258	0.040
1431	Serotransferrin	TF	79294.5	6.81	431	1.30358	0.032
1425	Protein ALEX	GNAS	68418.9	5.7	255	−1.84374	0.013
2303	retinol-binding protein4	RBP4	23301.3	5.77	257	−1.67608	0.011
1717	Keratin, cytoskeletal 9	KRT9	62254.9	5.14	272	−1.65378	0.018
2395	Keratin, cytoskeletal 1	KRT1	66170.1	5.97	163	−1.60070	0.027
2072	Isoform2 of vinculin	VCL	31750.2	8.1	127	−1.43492	0.044
849	Gelsolin	GSN	29106.8	7.71	56	−1.36868	0.039

MW:Molecular Weight; PI:Peptides Identified.

### Changes in APOA-I and GANS after risperidone treatment: ELISA

Plasma levels of APOA-I and GNAS were measured by ELISA for all 80 subjects before and after treatment. APOA-I was significantly increased and GNAS was significantly decreased after treatment (*p*'s<0.001); the results were consistent with the findings obtained from the proteomic analysis (Table 5).

**Table 5:** APOA-1 and GNAS measured by ELISA before and after 8 weeks of risperidone treatment (N = 80).

	Before treatment	After treatment	*t* value	*p* value
APOA-1 (mg/ml)	1.32±0.22	1.70±0.24	−6.77	<0.001
GNAS (ng/ml)	1.57±0.003	1.55±0.01	10.397	<0.001

### Relationships between changes in APOA-I, GNAS and changes in metabolic measures over 8 weeks of risperidone treatment

Significant positive relationships were found between changes in APOA-I and changes in triglycerides (*r* = 0.350, *p* = 0.001), total cholesterol (*r* = 0.319, *p* = 0.004), and BMI (*r* = 0.229, *p* = 0.041) over 8 weeks of risperidone treatment. No significant relationships were found between changes in APOA-1 and changes in fasting glucose LDL or HDL (*p*'s>0.05). After controlling for age, gender and family history of diabetes, the relationships between changes in APOA-I and changes in triglycerides, total cholesterol and BMI remain significant (*p*'s<0.05, Table 6). There were no significant relationships between changes in GNAS and changes in metabolic parameters (*p*'s>0.05). After controlling for age, gender and family history of diabetes, there was a trend positive relationship between changes in GNAS and changes in BMI (*r* = 0.211, *p* = 0.061, Table 6).

**Table 6:** Partial correlations between changes in APOA-I, GNAS and changes in metabolic measures after controlling for age, gender and family history of diabetes (N = 80).

	APOA-1-(ELISA)		GNAS-(ELISA)	
	*r*	*p*	*R*	*p*
BMI (kg/m^2^)	0.229	0.041	0.211	0.061
blood glucose (mmol/l)	0.149	0.186	0.176	0.119
triglyceride (mmol/l)	0.350	0.001	0.096	0.396
Total cholesterol (mmol/l)	0.319	0.004	0.093	0.413
LDL (mmol/l)	0.020	0.858	−0.177	0.116
HDL (mmol/l)	0.126	0.266	0.088	0.438

## Discussion

The APOA-I genes are clustered on chromosome 11q23 [12], which has been a locus of interest in schizophrenia research because it is near the dopamine 2 receptor (DRD2) gene [13]. To date, the role of APOA-I in schizophrenia is not yet well defined. Previous cross-sectional studies examined the levels of APOA-I in schizophrenia patients with conflicting results. Decreased plasma levels of APOA-I were found in schizophrenia [14]. Huang et al. [15] reported that APOA-I was down-regulated in the cerebral spinal fluid (CSF), liver, peripheral red blood cells and serum in schizophrenia patients. However, a more recent study revealed that APOA-I was up-regulated in the CSF of first episode patients with schizophrenia who were on antipsychotic treatment [16]. The heterogeneity of study samples and exposure to various antipsychotic medications may explain the inconsistency in previous findings.

In the present study, we found the plasma levels of APOA-I were significantly increased after 8 weeks of risperidone treatment in drug-naïve, first episode schizophrenia patients. This finding is consistent with a previous report in rats treated with antipsychotic medication haloperidol [17]. In addition, we found that, among 80 patients with schizophrenia, the increase in APOA-I after 8 weeks of risperidone treatment was associated with significant increases in BMI, triglyceride and total cholesterol, independent of age, gender, age of illness onset, family history diabetes. Our results are consistent with previous findings from various non-psychiatric populations [18]. APOA-I belongs to the apolipoprotein A1/A4/E family, which is produced in the liver and small intestine [19]. APOA-I participates in the reverse transport of cholesterol from tissues to the liver for excretion by promoting cholesterol efflux from tissues and by acting as a co-factor for the lecithin cholesterol acyltransferase (LCAT) [20]. APOA-I has been reported to play an important role in lipid transportation and metabolism [21]. Consequently the up-regulation of APOA-1 may result in increased plasma levels of cholesterols and possibly other metabolic disturbances.

GNAS was reported to be associated with obesity and insulin resistance [22]. The present study found a significant down-regulation of GNAS in schizophrenia patients after 8 weeks of risperidone treatment. However, the magnitude of change in GNAS (about 0.8%) should be interpreted with caution in the context of 8–10% ELISA assay precisions for GNAS. GNAS is part of Gα, which is a subunit of heterotrimeric G-proteins. Increased expression of G-α has been shown to activate the adenylyl cyclase signal transduction cascade, resulting in accumulation of the intracellular second messenger cyclic AMP (cAMP), a major player in the propoptotic process [23], [24]. Our study showed a trend positive relationship between changes in GNAS and changes in body mass index. Whether or not the down-regulation of GNAS is related to metabolic side effects of risperidone treatment is still unclear.

We examined the metabolic side effects of risperidone in our study as this medication is one of the most commonly used first-line antipsychotics used in first episode schizophrenia patients in China. One interesting question is whether or not there is a dose-response relationship with regard to risperidone's metabolic side effects, which could not be examined in our study given the narrow dose range (4–6 mg/day) used. Further, it would be interesting to examine the role of APOA-I and GNAS in first episode schizophrenia treated with those antipsychotic medications that have higher risk of metabolic side effects, such as olanzapine.

Most antipsychotic medications are associated with an increased risk for metabolic disturbances, such as weight gain, obesity, diabetes, hyperlipidemia, which ultimately contribute to an increased risk for cardiovascular disease in patients with schizophrenia [25]. So far there is a lack of reliable predictors for metabolic risk in individual patients who are on antipsychotic treatment. To our knowledge, the present study was the first to explore potential biomarkers related to metabolic side effects of antipsychotic treatment in first episode schizophrenia using proteomic analysis. Our results suggest that APOA-I could be a novel biomarker to predict metabolic side effects associated with risperidone treatment in first episode schizophrenia.

The study has several limitations: 1) Even though we were able to replicate the findings from a subgroup of patients (N = 15) in the entire study sample (N = 80), it is unclear if our findings can be replicated in a larger sample given the inherent lack of reproducibility in proteomic studies. 2) There was no correction for multiple testing in changes of plasma proteins after risperidone treatment; 3) Our study did not have a control group. The use of a more metabolically neutral antipsychotic medication as a control arm would help determine whether the findings in our study are specific to risperidone's metabolic impact. 4) The study time duration was relatively short. Despite the statistical significance in the changes of lipid measures after 8 weeks of risperidone treatment, the clinical significance of those changes was less evident; clinically meaningful changes in lipids usually take longer than 8 weeks to happen. 5) The generalizability of the findings is limited because only patients on risperidone were included in the study. Future studies with a larger sample size, a well-defined control group, a longer study time duration are need. APOA-I could be of great clinical utility if future studies confirm that it can predict antipsychotic-associated weight gain and other metabolic problems, especially in those patients who are on antipsychotic medications that carry a higher metabolic risk, such as olanzapine or clozapine.
